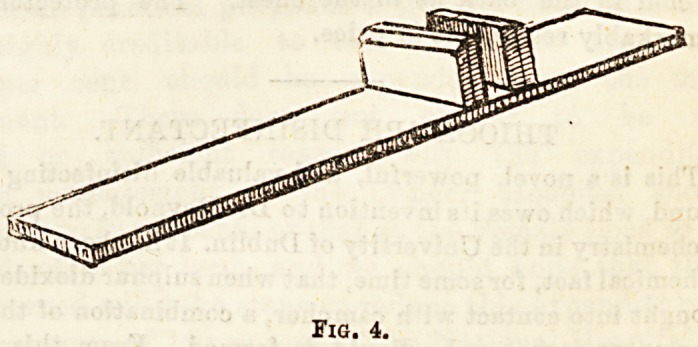# The Treatment of Fractures of the Shaft of the Femur

**Published:** 1892-10-15

**Authors:** 


### BRISTOL ROYAL INFIRMARY.

### The Treatment of Fractures of the Shaft of the Femur.

It is proposed to give in this article a short account of the most important practical points to be attended to in the treatment of an ordinary fracture of the shaft of the femur. Such cases more frequently come under the care of the hospital surgeon than of the general practitioner, therefore the details described, which are those carried out at the Bristol Royal Infirmary, may be of practical interest, though nothing new or unusual is claimed for the methods employed at this institution.

We must confine ourselves to the shaft of the bone, excluding the condyles, neck or trochanters. The place where the bone usually breaks is the middle third; and next in frequency the lower third; the upper part of the shaft is not so frequently broken, and in this last injury the tilting of the upper fragment often demands a different treatment from the one weare about to describe.

The special difficulty about all these cases is to overcome the traction of the muscles and to keep the bones in such a position as to prevent deformity. It has been attempted to surmount this in two ways, viz.: (1) By extending the leg in a straight position, and (2) by relaxing the muscles by slight flexion at the knee joint. This latter plan is useful in certain fractures, but in ordinary cases the general experience of hospital surgeons has come to be in favour of the former.

It is difficult to give directions as to the *undressing* of the patient, but this should be done without disturbing the horizontal position more than can be helped. It should be remembered that as the limb is to be kept in perfect rest for a long period, a thorough but gentle washing is advisable before any apparatus is applied.

The condition of the bed is all important. It is essential that it should be smooth and flat. This is arranged by the introduction of transverse boards on which a mattress, properly covered, is placed. Many contrivances have been used for the cleanly disposal of the excreta, such as circular holes in the mattress, &c., through which the fæces may pass into a pan below the bed. But it has been found that with ordinary care a flat bed-pan can be introduced without disarranging the fractured limb. The bedstead should be low, and narrow enough for the surgeon or nurse to easily attend to the patient from either side. There should be no footpiece, or only a low one, consisting of one transverse bar on which the extension pulley may be fastened. This latter consists of a

Fig. 1.

stout piece of wood (Fig. 1) oblong in shape, pierced in two places by the brass hooks *(h)*. These hooks, which fasten the apparatus to the transverse bar just referred to, have screws attached on the other side of the board *(s)*, so that their hold may be made tight and firm. On the top of the wooden block is the two-wheeled pulley *(p)*. The extending force consists of a bag *(b)*, into which weights may be placed. Where it is impossible to obtain this simple contrivance, a rough, but fairly effectual pulley may be made by putting an ordinary skewer through a cotton-reel and fastening the ends of the skewer to the upright s at the foot of the bed. (Fig. 2.)

Fig. 2.

The next detail is a very important one; it is the method of bringing the weight to bear upon the injured limb. This is effected as follows: A broad piece of stout strapping is fastened down the sides of the leg, and projects as a stirrup beyond the foot. It should reach up well above the knee, and should be well warmed, so that it may at once adhere firmly. It must be kept in position by three or four transverse strips arranged circularly round the leg. Unless these pieces of strapping are put on smoothly and made to adhere firmly, and if they are too narrow, the flesh will probably be cut by them and irritated. Into the loop of the stirrup is inserted a small oblong piece of thin wood with a hole in the centre, through which the extension cord is passed

Fig. 3.

and knotted. (Fig. 3.) The limb is to be then firmly bandaged as high as the fracture.

By this means the pull of the weight is upon the lower part of the thigh as well as upon the leg; but if the longitudinal pieces of strapping only reach to the knee, the extending force acts on the ligaments of that joint and materially weakens them.

Two other practical points must now be considered, viz., (1) how to keep the leg from lateral movement of any kind, and (2) how to apply some counter-extension to the weight. The first of these is sometimes carried out by placing sand bags on either side of the limb, but the most usual practice at the Bristol Infirmary is to put a Liston's splint down the outer side of the leg, and bandage it firmly to the injured member below the knee and as high as the fracture, with a few turns round the patient's body. It is found that this does not at all interfere with the extension. A sand bag may be applied to the inside of the leg, or the following contrivance may be used: A narrow piece of wood about four-

Fig. 4.

teen inches long and four wide is provided with two transverse slips fastened on at right angles, as depicted in Fig. 4. These should not be at the centre of the long piece, but nearer one end (the side of the fracture). Into the small groove made by the two upright blocks the Liston's splint may be inserted at the patient's ankle. This effectually steadies the long splint, and does not interfere with the extending force; but it is important that the interval between the two small blocks should be sufficient to allow a gliding of the splint, and at the same time narrow enough to prevent any "wobbling." If it is firmly fastened to the Liston it materially interferes with extension. Having obtained the two requisites of extension combined with fixation, we must now deal with counter-extension. The perinæal band is very rarely used now on account of its unpleasant friction, and the excoriation it is so apt to produce. Instead of this the foot of the bed is raised by substantial blocks of wood placed under the castors. These blocks are shaped like a truncated cone, with a small hollow at the top, and can easily be made. Other contrivances may be used, but whatever is employed there must be absolutely no chance of slipping.

The above methods may be modified in individual cases, but they will be found to fulfil the required conditions; that is, extension, counter-extension, and fixation. They are easy to apply, comfortable, and the results obtained are satisfactory. The after treatment of these fractures is by plaster of Paris bandages, which may be used when the bone has sufficiently united to bear passive movement without injury.

## Figures and Tables

**Fig. 1. f1:**
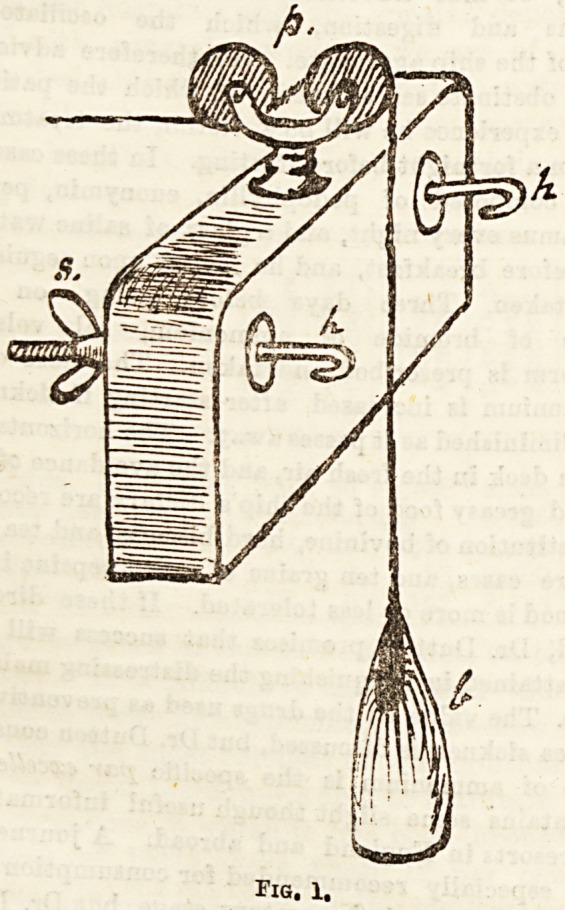


**Fig. 2. f2:**
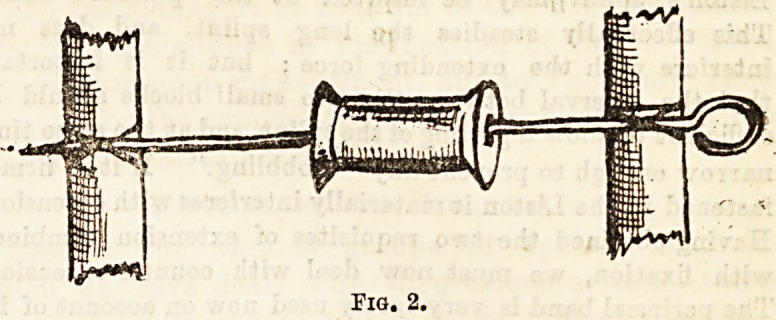


**Fig. 3. f3:**
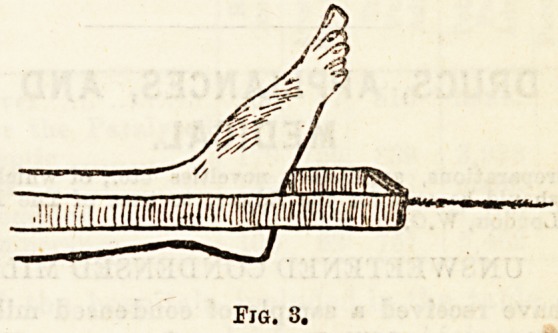


**Fig. 4. f4:**